# A Basic Model to Predict Enteric Methane Emission from Dairy Cows and Its Application to Update Operational Models for the National Inventory in Norway

**DOI:** 10.3390/ani11071891

**Published:** 2021-06-25

**Authors:** Puchun Niu, Angela Schwarm, Helge Bonesmo, Alemayehu Kidane, Bente Aspeholen Åby, Tonje Marie Storlien, Michael Kreuzer, Clementina Alvarez, Jon Kristian Sommerseth, Egil Prestløkken

**Affiliations:** 1 Department of Animal and Aquacultural Sciences, Norwegian University of Life Sciences, 1432 Ås, Norway; puchun.niu@nmbu.no (P.N.); Alemayes@nmbu.no (A.K.); bente.aby@nmbu.no (B.A.Å.); maria.clementina.alvarez.flores@nmbu.no (C.A.); egil.prestlokken@nmbu.no (E.P.); 2 Norwegian Institute for Bioeconomy (NIBIO), 7031 Trondheim, Norway; helge.bonesmo@nibio.no; 3 Tine SA, 1430 Ås, Norway; Tonje.Marie.Storlien@tine.no (T.M.S.); jon.kristian.sommerseth@tine.no (J.K.S.); 4 ETH Zurich, Institute of Agricultural Sciences, 8092 Zurich, Switzerland; michael.kreuzer@usys.ethz.ch

**Keywords:** dairy cattle, prediction model, methane conversion factor, dry matter intake, fatty acid, neutral detergent fiber

## Abstract

**Simple Summary:**

Many techniques exist to quantify enteric methane (CH_4_) emissions from dairy cows. Since measurement on the entire national cow populations is not possible, it is necessary to use estimates for national inventory reporting. This study aimed to develop (1) a basic equation of enteric CH_4_ emissions from individual animals based on feed intake and nutrient contents of the diet, and (2) to update the operational way of calculation used in the Norwegian National Inventory Report based on milk yield and concentrate share of the diet. An international database containing recently published data was used for this updating process. By this the accuracy of the CH_4_ production estimates included in the national inventory was improved.

**Abstract:**

The aim of this study was to develop a basic model to predict enteric methane emission from dairy cows and to update operational calculations for the national inventory in Norway. Development of basic models utilized information that is available only from feeding experiments. Basic models were developed using a database with 63 treatment means from 19 studies and were evaluated against an external database (*n* = 36, from 10 studies) along with other extant models. In total, the basic model database included 99 treatment means from 29 studies with records for enteric CH_4_ production (MJ/day), dry matter intake (DMI) and dietary nutrient composition. When evaluated by low root mean square prediction errors and high concordance correlation coefficients, the developed basic models that included DMI, dietary concentrations of fatty acids and neutral detergent fiber performed slightly better in predicting CH_4_ emissions than extant models. In order to propose country-specific values for the CH_4_ conversion factor *Y*_m_ (% of gross energy intake partitioned into CH_4_) and thus to be able to carry out the national inventory for Norway, the existing operational model was updated for the prediction of *Y*_m_ over a wide range of feeding situations. A simulated operational database containing CH_4_ production (predicted by the basic model), feed intake and composition, *Y*_m_ and gross energy intake (GEI), in addition to the predictor variables energy corrected milk yield and dietary concentrate share were used to develop an operational model. Input values of *Y*_m_ were updated based on the results from the basic models. The predicted *Y*_m_ ranged from 6.22 to 6.72%. In conclusion, the prediction accuracy of CH_4_ production from dairy cows was improved with the help of newly published data, which enabled an update of the operational model for calculating the national inventory of CH_4_ in Norway.

## 1. Introduction

The increase in global average surface temperature over the past half-century cannot be fully explained by natural climate variability. Scientific evidence indicates that the leading cause of climate change in the most recent half century is anthropogenic. Especially damaging is the increase in the concentration of atmospheric greenhouse gases (GHG), including carbon dioxide (CO_2_), chlorofluorocarbons (CFCs), methane (CH_4_), tropospheric ozone and nitrous oxide (N_2_O) [1]. Animal husbandry is a source of anthropogenic GHG emission with CH_4_ and N_2_O as main gases, accounting for 30% of the total emissions by the agricultural sector [2]. Through CH_4_, dairy production systems contribute, expressed in CO_2_-equivalents, approximately one-half of the GHG emissions attributed to animal husbandry. Of this, on average 81% originate from enteric fermentation and 19% from manure [3]. Enteric CH_4_ arises mainly as a side-product from rumen microbial fermentation of feed, especially fiber, to volatile fatty acids (VFAs). This fermentation process generates an excess of hydrogen (H_2_) that is removed in the rumen by methanogens through reduction of CO_2_ to CH_4_.

The factors determining the amount of enteric CH_4_ produced per animal include feed dry matter intake, diet composition (e.g., contents of ether extract (EE) or fatty acids (FAs) and neutral detergent fiber (NDF)), rumen microbial population, host physiology and host genetics [4]. To identify efficient mitigation strategies, the amount of CH_4_ produced by the dairy system needs to be quantified as accurately as possible. Direct measurements of enteric CH_4_ production (MJ/day) from cattle can be conducted using various methods, such as respiration chambers, sulfur hexafluoride (SF_6_) tracer technique and the GreenFeed (GF) system (C-Lock Inc., Rapid City, SD, USA; [5]). However, when the total national CH_4_ emissions need to be assessed for an inventory these techniques are not feasible due to the sheer number of measurements which would be needed. For this purpose, often quantitative approaches such as empirical modelling have been used to estimate CH_4_ production in dairy cows [6,7].

Accurate information about feed intake and dietary composition is required for good prediction but this information is available only from feeding experiments and thus for a limited number of animals, while information about milk yield and dietary concentrate share is available for the Norwegian dairy cow population from the Dairy Herd Recording System (TINE SA, Oslo, Norway) for a continuous time series starting in 1990 [8]. Thus, the present study involved the development of an accurate basic model for prediction of enteric CH_4_ production, and operational models for prediction of the CH_4_ conversion factor (*Y*_m_, % of gross energy intake (GEI) lost as CH_4_). The *Y*_m_ is globally used for national GHG emission inventories and research on mitigation strategies [9]. Previously, Nielsen et al. [6] published in 2013 a basic model for the prediction of enteric CH_4_ emission from dairy cows based on 47 treatment means from 12 studies. This equation is used in the Nordic Feed Evaluation System—NorFor [8]. One year later, Storlien et al. [7] developed another basic model based on 78 treatment means from 21 studies. This later model [7], and an operational model [8] using information about milk yield and concentrate share, are those which were used by the Norwegian Environment Agency (Miljødirektoratet) for the National Inventory Report to the United Nations Framework Convention on Climate Change (UNFCCC) and Kyoto Protocol/Paris Agreement. The operational model is dependent on the output of CH_4_ production predicted by the basic model. The basic model [7] was developed based only on studies published until 2013. In addition, this model did not take into account the effect of dietary NDF.

Therefore the objectives of the present study were (1) to extend the database of Storlien et al. [7] with more recent studies; (2) to develop basic models using this extended database, and evaluate them against extant models in their performance in predicting enteric CH_4_ production; (3) to use our best performing basic model to predict CH_4_ production and to calculate *Y*_m_ with the help of the NorFor feed analysis database (NorFor-database) [8]; and (4) to update operational models where energy-corrected milk (ECM) and dietary concentrate share in the diet were used to predict *Y*_m_ and GEI, respectively.

## 2. Materials and Methods

The basic models were developed using information of CH_4_ production, dry matter intake (DMI) and dietary nutrient compositions, from published feeding experiments. The operational model was developed to predict *Y*_m_ using energy corrected milk and dietary concentrate share based on an operational database (NorFor) [8] simulated to cover a wide range of feeding situations reported in the Dairy Herd Recording System (TINE SA, Oslo, Norway).

### 2.1. Basic Model Database

The basic model database originally used by Storlien et al. [7] was collated from 21 studies (Nordic, European, intercontinental) published from 1997 to 2013, consisting of 78 treatment means. The database was divided into two subsets, one for model development (*n* = 42) and one for model evaluation (*n* = 36). In the present study, the subset for basic model development from Storlien et al. [7] was extended by adding data published since 2013 where CH_4_ production, forage proportion, DMI and contents of EE or FAs and NDF in diets for dairy cows were reported (*n* = 21 treatment means from 8 studies, highlighted in grey shading in Table 1; Nordic, European and intercontinental origin). Treatments investigating impact of feed additives were excluded from the dataset, except for those based on terrestrial plant lipids which are commonly used in dairy cows’ diet and are frequently represented in the database. The resulting database (*n* = 99, from 29 studies on dairy cows) is described in Table 1, where roughage and concentrate ratio and CH_4_ production along with corresponding DMI are presented. The roughage was mainly comprised of silage from grass, maize and alfalfa, while barley, maize and soybean meal were the main ingredients of the concentrates. The CH_4_ production was determined by the sulfur hexafluoride (SF_6_) gas tracer technique in 14 studies, by respiration chambers in 13 studies, by the hood calorimetry technique in one study and by the GreenFeed system in one study.

### 2.2. Development of Basic Models

CH_4_ production was predicted by fitting mixed models to the lmer [39] procedure of R statistical language (R Core Team 2016; version 4.0.2) (Equation (1)):

(1) $$Y=\beta_{0}+\beta_{1}X_{1}+\beta_{2}X_{2}+\beta_{n}X_{n}+R_{j}+\varepsilon$$

where Y denotes the response variable of CH_4_ production, $\beta_{0}$ denotes the fixed effect of intercept; $X_{1}$ to $X_{n}$ denote the fixed effects of predictor variables and $\beta_{1}$ to $\beta_{n}$ are the corresponding slopes; $R_{j}$ denotes the random study effects of the experiment; ε denotes the within-experiment error. To account for differing accuracy in observed means, models were fitted using the WEIGHT statement in R (R Foundation for Statistical Computing, Vienna, Austria), where the data were weighted according to the number of observations [40]. The effect of the categorical factor CH_4_ measurement techniques (tracer gas, chamber, headhood, GF) was included in the model as a fixed effect prior to final model development and found to be not significant (*p* > 0.1), and thus was not incorporated in the final models fitted. The presence of multicollinearity of fitted models was examined based on the variance inflation factor (VIF). A VIF in excess of 5 was considered an indicator of multicollinearity [41]. Multicollinearity was not detected. All parameters included in the developed models presented were significant at *p* < 0.05.

### 2.3. Basic Model Evaluation

In total, ten models were evaluated, including three models developed in the present study and seven extant models with similar input variables (DMI and dietary nutrient contents). The models were compared through assessing their abilities of predicting CH_4_ production, using mean squared prediction error (MSPE) and concordance correlation coefficient (CCC). The MSPE was calculated according to Bibby and Toutenburg [42] as shown in Equation (2):

(2) $$MSPE=\frac{\sum_{i=1}^{n}{\left(Y_{i}-{\hat{Y}}_{i}\right)}^{2}}{n}$$

where $Y_{i}$ denotes the observed value of the response variable for the ith observation, ${\hat{Y}}_{i}\;$ denotes the predicted value of the response variable for the ith observation, n denotes the number of observations. The root mean square prediction error (RMSPE) was used to assess overall model prediction accuracy because its output was in the same unit as the observations. In the present study,  RMSPE  was reported as a proportion of observed CH_4_ production means in order to compare the predictive capability of models with different predicted means. A smaller RMSPE implies a better model performance. The MSPE was decomposed into error in central tendency (ECT), error due to disturbance (ED) or random error and error due to regression (ER).

The ECT, ED and ER fractions of MSPE were calculated as follows:

(3) $$ECT={\left(\bar{P}-\bar{O}\right)}^{2}$$

(4) $$ED=\left(1-R^{2}\right)\times S_{o}^{2}\;$$

(5) $$ER={\left(S_{p}-R\times S_{o}\right)}^{2}\;$$

where $\bar{P}$ and $\bar{O}$ are the predicted and observed means, $S_{p}$ is the predicted standard deviation, $S_{o}$ is the observed standard deviation and R is the Pearson correlation coefficient.

According to Lawrence and Lin [43], CCC is the product of a bias correction factor as the measurement of accuracy ($C_{b}$) and the precision measurement of Pearson correlation coefficient (r). The CCC was calculated as shown in Equation (6):

(6) $$\;CCC=r\times C_{b}$$

where:

Cb=[(v+1)/(v+µ^2)/2]−1v=So/Spµ=(P¯−O¯)/(SoSp)1/2

where $\bar{P}$, $\bar{O}$, $S_{o}$ and $S_{p}$ were defined above, and v indicates a measure of scale shift, and µ indicates a measure of location shift. The CCC evaluates the degree of deviation of the best-fit line from the identity line (y=x), and thus, the CCC of a model that is closer to 1, is an indication of better model performance.

### 2.4. Update of Operational Models

The operational equation from Storlien and Harstad [44] presently used for predicting *Y*_m_ was based on calculations in NorFor (Table 2), using intervals of 500 kg from 5000 to 12,000 kg of ECM. The Norfor database with CH_4_ production (not shown) predicted by the basic models, GEI and *Y*_m_ (not shown; calculated based on CH_4_ production and GEI) was used in the present study for the update of operational models. The standardized lactation curves in NorFor were employed to predict animal requirement for ECM production through the lactation cycle. Daily DMI was calculated for every second lactation week for each 500 kg interval of the 305-day lactation. Feed energy (GE, metabolizable energy (ME) and net energy (NE)), animal energy requirements and energy supplementation were calculated based on the Dutch net energy lactation (NEL) system [45] as modified by NorFor [8].

The data predicts standard feed rations during a 305-day lactation at different lactation yield, using three different forage qualities (Table 3), 5.7, 6.1 and 7.0 MJ NEL per kg DM, representing low, medium and very high energy content, respectively. Three complimentary concentrate mixtures, which are representative of what is used in practical diet formulation in Norway, were used in the diet formulation to meet the animal energy requirement (Table 3).

To observe the effects of different basic models on the output of operational models, the basic model that performed the best in predicting CH_4_ production, and models from Storlien et al. [7] and Nielsen et al. [6] were selected to predict CH_4_ production, respectively, and thus to calculate *Y*_m_ in the NorFor-database. Three operational models were therefore developed, in which the response variable was *Y*_m_, and the input variables were ECM and concentrate share in the diet. Moreover, GEI was also predicted with the same input variables. The *Y*_m_ and GEI were estimated by fitting a mixed effect model using the lmer [40] procedure of R statistical language (R Core Team 2016; version 4.0.2). The model employed is shown in Equation (7):

(7) $$Y=b_{0}+b_{1}X_{1}+b_{2}X_{2}+b_{n}X_{n}+S_{j}+\epsilon$$

where Y denotes the response variable of *Y*_m_ or GEI, $b_{0}\;$ denotes the fixed effect of intercept; $X_{1}$ to $X_{n}$ denote the fixed effects of predictor variables and $b_{1}$ to $b_{n}$ are the corresponding slopes; $S_{j}$ denotes the repeated effect of days after lactation at each ECM production level; ϵ denotes the error within a lactation cycle. The presence of multicollinearity of fitted models was examined based on the VIF. A VIF in excess of 5 was considered an indicator of multicollinearity [41]. Multicollinearity was not detected. The following equation was used to calculate the CH_4_ emission factor (EF) for 365 days, which can be used for estimating national CH_4_ emissions when the number of animals is known:

(8) $$\mathrm{EF}\;=\left(\mathrm{GEI}\;\cdot \;Y_{m}\;\cdot \;365\;\mathrm{days}/\mathrm{yr}\right)/55.65\;\mathrm{MJ}/{\mathrm{kg}\;\mathrm{CH}}_{4}$$

where EF denotes emission factor (kg CH_4_/head/year); GEI denotes gross energy intake (MJ/head/day); *Y*_m_ denotes CH_4_ conversion rate, which is the fraction of gross energy in feed converted to CH_4_.

## 3. Results

### 3.1. Development and Evaluation of Basic Models

Models 1, 2 and 3, which were developed in the present study, and other extant models, are presented in Table 4 with results of model evaluations. The models were arranged in descending order of CCC. Overall, the developed models and models from Storlien et al. [7] and Nielsen et al. [6] performed better than other extant models with respect to prediction accuracy (RMSPE and CCC), except that the lowest RMSPE was found in one of the models from Niu et al. [9] yet with low CCC. The overall performance of the extant models using only DMI as input variable did not perform as good as models where dietary FAs and/or NDF were included as input variables in addition to DMI. Model 1 slightly outperformed the model from Storlien et al. [7], judged by RMSPE (15.0 versus 15.3), owing to smaller ER. When NDF together with DMI and FAs was included as input variables in the models, evaluation through CCC and RMSPE indicated that model performances were improved (Model 2 and 3, as well as the Nielsen et al. [6] model). Model 2 and 3 performed even better, indicated by lower RMSPE and higher CCC, compared to the Nielsen et al. [6] model. It was assumed that cows are not emitting nor inhaling CH_4_ if they are not eating, hence the intercept was forced to zero in Model 2 to have Model 3 developed. The performance was somewhat compromised for Model 3 as compared to Model 2 mainly due to increased ED (Table 4).

**Figure 1:**
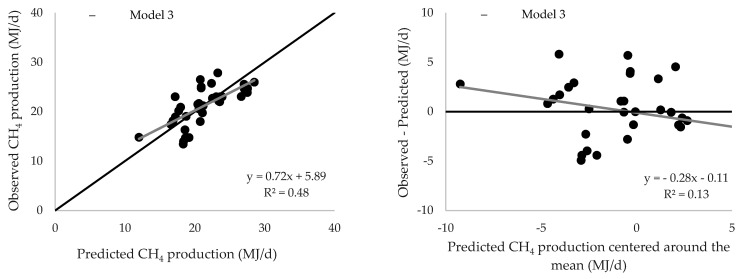

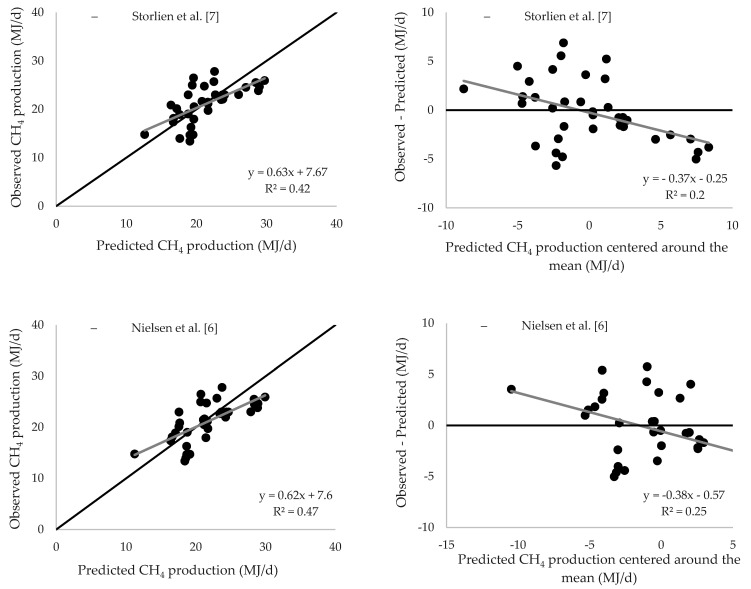
Observed versus predicted values of enteric CH_4_ production and the residuals (observed minus predicted) for basic models used in Norway and the Model 3 developed in the present study. The graphs to the left show that the models overestimate CH_4_ emissions at the lower range and underestimate emissions at the upper range. The graphs to the right show the presence of a linear bias (slope) and the presence of a mean bias (intercept).

Plots of observed versus predicted values of enteric CH_4_ production and the residuals (observed minus predicted) for Model 3 and models from Storlien et al. [7] and Nielsen et al. [6] are presented in Figure 1. These three models were selected to calculate CH_4_ production in the NorFor-database, respectively.

### 3.2. Update of Operational Models

The operational models for the prediction of *Y*_m_ and GEI are presented in Table 5. There was a significant positive relationship between GEI and both ECM and concentrate share. When estimating *Y*_m_, both predictor variables were negatively correlated to the response variable.

Table 5 shows the annual production of CH_4_ assuming an annual milk yield of 6000, 8000 and 10,000 kg ECM and an averaged concentrate share of 38.0, 43.5 and 50.0%, respectively. These are typical concentrate shares in Norway where concentrate is used on all dairy farms. When milk yield and concentrate share were increased, *Y*_m_ was predicted to decrease in all models, whereas GEI and the CH_4_ emission factor were predicted and calculated to increase, respectively. At a production level of 6000 kg ECM and a 38% concentrate share, when the prediction of *Y*_m_ was obtained through the model from Storlien et al. [7], the prediction of *Y*_m(S)_ (see footnote to Table 5) and the CH_4_ emission factor (127.7 kg/year per cow) were the lowest. On the contrary, using the model from Nielsen et al. [6] to predict CH_4_ production and *Y*_m_ under the same conditions with the NorFor-database led to the highest predicted values of both *Y*_m(N)_ (see footnotes to Table 5) and the CH_4_ emission factor. The same ranking for both *Y*_m_ and the CH_4_ emission factor was found at a production level of 8000 kg ECM and a 43.5% concentrate share, while the differences among predictions of *Y*_m(S)_, *Y*_m(M)_ (see footnotes to Table 5) and *Y*_m(N)_ were decreased. At a production level of 10,000 kg ECM and a 50% concentrate share, predictions of *Y*_m(M)_ and correspondingly the CH_4_ emission factor were the lowest, which were 6.22 and 163.7 kg/year per cow, respectively.

## 4. Discussion

The aims of the present study were to develop a basic model which can be used as a method for the accurate calculation of enteric CH_4_ emissions from individual dairy cows, and to update the existing operational model for the prediction of *Y*_m_ and the CH_4_ emission factor to be used in the national GHG inventory in Norway.

### 4.1. Relationship between Methane Production and Dietary Factors in the Basic Models

In the present study, DMI and dietary concentrations of FAs and NDF were used and confirmed as key predictor variables for CH_4_ production in dairy cows. DMI was the most important variable for the prediction of enteric CH_4_ production in all models evaluated. The significant positive relationship is consistent with the knowledge that CH_4_ production increases with feed intake due to the greater availability of substrate for microbial fermentation [8,48,49]. A linear relationship between DMI and CH_4_ production has been observed in many studies [6,7,46]. However, an increased intake potentially increases passage rate of feed through the rumen, resulting in a decline in rumen fermentation and CH_4_ production per unit of feed [50]. Subsequently, the percentage of gross energy lost as CH_4_ declines [9], but at the same time digestibility may decline resulting in an unchanged methane emission intensity per unit of milk or meat produced. Nevertheless, the first assumption implies that in theory a model of CH_4_ production based on DMI, GEI or MEI, should be nonlinear [8]. The only nonlinear model [47] that was evaluated in the present study did not perform as robust as others, which may be due to that only feed intake was accounted for in their model. This could be justified by Bell et al. [51], where the residual variation (difference between observed and predicted values) in CH_4_ emission was notably reduced after incorporating the significant fixed effects of dietary characteristics on CH_4_ yield, in addition to the effect of feeding level.

Fat content was the second most important variable for the prediction of enteric CH_4_ production in all models evaluated. In the present study, the accuracy of prediction was better with the inclusion of dietary fat content in the equation compared to extant models where only DMI was used, and there was a significant negative relationship between fat and CH_4_ production. This was facilitated by not excluding experiments where fat had been supplemented. Indeed, CH_4_ production decreases through fat supplementation in the diet, as reviewed and studied by several groups [11,34,51]. The mode of action of fat on CH_4_ mitigation has been extensively studied [52]. The effect is based on the following components. (1) Biohydrogenation of unsaturated fatty acids utilizes H_2_ available for CH_4_ production. However, the complete biohydrogenation of one mol of linoleic acid can reduce CH_4_ production only by one mol and thus this is not quantitatively important [47]. (2) As fat is not fermentable, part of the reduced CH_4_ production with increased dietary fat concentration can be accredited to decreased supply of fermentable substrate for the microorganisms, also reducing hydrogen production [53]. (3) The most important component is a direct toxicity of fatty acids, especially that of lauric and myristic acid and polyunsaturated fatty acids, exhibiting against the archaeal methanogens [54]. (4) Finally, dietary fat concentration directly influences rumen fermentation by favoring propionate production at a cost of acetate or butyrate, or both, because protozoa are inhibited as well which results in declines in fiber digestion and hydrogen supply [55].

The accuracy of prediction was further improved when dietary NDF content was included in the equations along with DMI and fat, and there was a significant positive relationship between NDF and CH_4_ production as expected from earlier studies [6,56]. Studies focusing on the effect of different types of carbohydrates, indicate that high concentrations of starch and sugar (non-fibrous carbohydrates) increase the production of propionate but decrease that of acetate and butyrate, and the opposite is true for NDF (fibrous carbohydrates) [53,56]. The CH_4_ production is thus related to the VFA profile in such a way that higher NDF increases CH_4_ production by shifting short chain fatty acid proportion towards acetate which is associated with a higher hydrogen release [57]. The NDF content was only the third most important variable for the prediction of enteric CH_4_ production in all models evaluated, i.e., the influence of NDF content was less pronounced than that of fat contents.

Model 3 was developed from Model 2 by applying biologically sensible constraints, e.g., zero CH_4_ at zero intake [8]. In the current study, Model 3 was selected based on model performance as the updated model over models from Nielsen et al. [6] and Storlien et al. [7]. Different from the Storlien et al. [7] equation, Model 3 allows for considering effects of NDF concentration in the feed in addition to fat concentration. The concentration of NDF will vary with forage proportion and quality in the diet. A positive coefficient for NDF reflected reduced CH_4_ production by earlier harvesting of grass for silage as NDF concentration in grass increases with harvesting time. Model 3 has the same input variables as the Nielsen et al. [6] equation but yields slightly lower estimates of the comparatively high CH_4_ emission factor in Norway (Table 5).

### 4.2. Update of Operational Models

The NorFor-database applied in the present approach is the same as used by Storlien and Harstad [44], and the calculation of GEI remained unchanged. No major changes in milk yield and quality of silage and concentrate have taken place since 2015 (pers. com. TINE and Felleskjøpet Fôrutvikling), and therefore, it was considered unnecessary to recalculate the NorFor-data, except CH_4_ production. However, since input data of predicted enteric CH_4_ production was changed, equations for prediction of *Y*_m_ based on ECM and concentrate share also changed. Many studies have suggested using factors such as fiber digestion [58,59] and dietary lipid content [60], either as the single or multiple variables of a *Y*_m_ model. However, in the present study a country-specific approach was used for the prediction of *Y*_m_ using the same method as Storlien and Harstad [44]. This approach allows country-specific information to be included in the development of equations without access to data that are not readily available, such as fiber and lipid contents in the diet. In the Norwegian cow recording system (CRS) individual milk yield and concentrate supplementation is reported 11 times per cow per year, and data from 1.16 million individual cow observations are available [8]. The recorded information in the Norwegian CRS was not directly included for updating the operational models. Instead, the simulated Norfor-database (Table 2) included a variety of variables such as feed intake and composition, *Y*_m_ and GEI, in addition to milk yield and concentrate share. In order to develop representative *Y*_m_ for the about 200,000 Norwegian dairy cows this was essential for being able to take into account the effect of dietary composition and the experiments using grass-based diets, which were considered when updating CH_4_ production in the NorFor-database. From Table 5 the predicted *Y*_m_, depending on the level of production, ranged from 6.22 to 6.72%, which is within the range of the IPCC default *Y*_m_ of 6.5% ± 1% [61]. This default value is recommended by IPCC [61] for all types of cattle and buffalo, except feedlot cattle fed at least 90% concentrate. However, the lowest predicted value 6.22% was yet higher than that given by Hellwing et al. [62] for Danish dairy cows, which was 6.02% and 5.98% of GE intake for Holstein and Jersey cows, respectively. Accordingly, Lesschen et al. [63] concluded that within the EU countries, the GHG emission per kilogram milk produced was lowest in Denmark. In the Netherlands, a Tier 3 approach which addresses effects of nutritional details on enteric CH_4_ emission is used for the national inventory, with a predicted CH_4_ emission factor in a smaller range of 110.5 to 129.4 kg/cow/year and a lower predicted *Y*_m_ of 5.88% to 6.07% of GE intake [64] at unspecified production level. In France, a new equation was developed to predict enteric CH_4_ that complies with IPCC rules for a Tier 3 method and is based on digestible organic matter intake (DOMI). The representative dairy cow of 650 kg BW and 6300 kg annual milk yield was estimated to produce only 119.3 kg CH_4_/year using a default *Y*_m_ value of 6.50% [65], while the operational model of the present study yields as much as 130 kg CH_4_ per year at a production level of 6000 kg ECM/year. The discrepancies across countries can possibly be explained by differences in diet composition, as there is a higher dietary proportion of forage in Norway, and milk yield is moderate compared to other European countries and USA. With increasing milk yield and concentrate share, *Y*_m_ decreases, whereas the CH_4_ emission factor increases. This is due to the fact that more energy is allocated to milk production, as the CH_4_ emission in kg per kg ECM decreased. These results are in accordance with those reported by Kirchgessner [66] and Volden and Nes [8]. Accordingly, CH_4_ emission decreases by 2.8 g/kg milk and 41.4% of total CH_4_/milk per day when milk production is increased from 4000 to 6000 kg and from 5000 to 9000 kg, respectively.

The value of operational models is dependent on correct and annually updated reporting of average annual milk yield and concentrate share of dry matter intake. In addition, an updated basic model could help refining the estimates of CH_4_ production, which could ultimately improve the estimate of *Y*_m_. As discussed above, it is possible by using the above information to develop a robust model for use in Norway for the calculation of enteric CH_4_ emission from dairy cows. Further, the recommended equation is well suited for improving the CH_4_ emissions estimates of the farm level net GHG model HolosNor [67]. The HolosNor is used as an advisory tool [68], and the implementation of Model 3 developed in the current work will be helpful for quantifying and advising mitigation strategies at farm level. In the current models developed, the effects of dietary changes were considered only indirectly through calculation of *Y*_m_ using basic models. Therefore, a further improvement in the prediction accuracy might be expected for a tier 3 model that includes also a dynamic and mechanistic model of fermentation biochemistry to calculate enteric CH_4_ emission inventories [64,65].

## 5. Conclusions

Three basic models were developed in this study. Among them, Model 3 with input variables of DMI, dietary concentrations of FAs and NDF, turned out to predict CH_4_ production more accurately than the extant models from Nielsen et al. [6] and Storlien et al. [7]. Using a basic model database containing recently published data improved CH_4_ production estimates in the operational model. Hence, this basic (Model 3) and updated operational equation for calculation of enteric CH_4_ emission from individual dairy cows in Norway is now used by the Norwegian Environment Agency (Miljødirektoratet). This is essential to improve accuracy of carbon footprint assessment of dairy cattle production systems and to help quantify and communicate effective mitigation strategies.

## Figures and Tables

**Table 1:** Summary of database for the basic models.

Data-Base ^a^	Stage ^b^	N ^c^	Roughage	Concentrate	Forage Proportion (% of DM)	DMI (kg/day) ^d^	CH_4_ Collection Technique ^e^	CH_4_ (MJ/day) ^f^	References
D	L	4	Maize silage	Ground maize	50	20	1	20 (14–26)	[10]
D	NL	4	Grass hay or barley silage	Barley grain	95	11	1	12 (11–17)	[11]
D	L	3	Grass silage	Oats, barley, peas and rapeseed cake	69	16	1	17 (16–18)	[12]
D	L	2	Grass silage	Barley, wheat and maize	73	23	1	32 (28–36)	[13]
D	L	3	Grass silage	Barley, wheat and oats	77	20	1	26 (24–28)	[14]
D	L	6	Ryegrass, white and red clover	Pelleted barley	77	19	2	24 (23–26)	[15]
D	L	3	Grass and maize silage	Barley	67	17	2	19 (17–21)	[16]
D	L	3	Alfalfa hay and alfalfa silage	Barley, maize and peas	51	26	1	23 (22–25)	[17]
D	L	4	Grass silage	Barley	70	17	1	25 (21–30)	[18]
D	NL	4	Grass silage	Wheat starch (non-NDF concentrate)	83	8	1	11 (10–12)	[19]
D	L	6	Grass silage	Wheat starch (non-NDF concentrate)	69	15	1	18 (17–19)	[20]
D *	L	4	Grass silage	Oats, barley and rye	50	19	1	26 (25–28)	[21]
D *	L	2	Rye grass, white clover or mature diverse pasture	0	100	21	4	27 (26–28)	[22]
D *	L	1	Grass clover silage	0	100	12	2	17	[23]
D *	L	1	Maize, grass/clover silage	Barley, sugar beet pulp and rapeseed cake	50	19	2	18 (16–20)	[24]
D *	L	2	Hay, maize silage and grass pellets	Wheat, maize, barley, rapeseed cake	80	21	2	27 (26–28)	[25,26]
D *	L	2	Maize and grass/clover silage	Whole cracked rapeseed	55	21	2	25 (23–27)	[27]
D *	L	6	Maize, grass silage and hay	Oat, soybean, wheat and apple pulp	50	17	2	22 (18–25)	[3]
D *	L	3	Ryegrass	0	100	15	2	17 (16–19)	[28]
E	L	4	Grass and maize silage	Rapeseed meal, rapeseed cake, cracked rapeseed	51	18	1	20 (17–23)	[29]
E	L	6	Grass silage and maize silage	Rapeseed meal, whole crushed rapeseed	64	17	1	20 (18–22)	[30]
E	L	4	Alfalfa hay and ryegrass silage	Cracked wheat grain	63	20	2	26 (25–28)	[31]
E	L	2	Maize and grass silage	Soybean meal and rolled barley	80	17	1	18 (14–22)	[32]
E	L	2	Maize silage and alfalfa haylage	Cracked wheat grain	67	16	1	23 (21–25)	[33]
E	L	4	Barley silage	Steam rolled barley and pelleted supplement	45	18	2	15 (13–16)	[34]
E	L	2	Haylage, maize silage and high moisture maize	Maize gluten and soybean meal	59	15	3	19 (15–23)	[35]
E	L	4	Hay, grass and maize silage	Barley and wheat bran	75	17	2	22 (18–24)	[36]
E	L	4	Maize and grass silage	Rapeseed meal, sunflower meal, ground wheat and maize gluten feed	56	20	2	23 (22–23)	[37]
E	L	4	Alfalfa silage	High moisture maize and dry maize	88	24	2	25 (24–26)	[38]

^a^ D, experiments used for model development; * indicates newly added studies; E, experiments used for model evaluation; ^b^ Physiological stage defined as either lactating (L) or non-lactating (NL); ^c^ Number of treatment means in study; ^d^ Mean value of dry matter intake (DMI) for experiment; ^e^ 1, tracer gas technique; 2, chamber; 3, head hood; 4, GreenFeed system; ^f^ Mean (min–max) value for experiment; the following factors were used in converting CH_4_ in L/day to g/day and g/day to MJ/day: 1 L CH_4_ = 0.716 g; 1 g CH_4_ = 0.05565 MJ.

**Table 2:** Mean (min-max) value of concentrate share, dry matter intake (DMI) and gross energy intake (GEI) throughout a 305-day lactation with various combinations of silages and concentrates at different levels of energy corrected milk (ECM) production ^a^ in the NorFor-database used for the operational models.

Yield (ECM, kg)	Silage ^b^	Concentrate ^c^	Concentrate Share, % DM ^d^	DMI, kg/d	GEI, MJ/day
5000	1	I	11 (0–37)	15 (12–17)	279 (232–312)
	2	II	20 (0–53)	15 (12–17)	282 (228–327)
	3	II	25 (0–50)	16 (12–18)	292 (233–340)
5500	1	III	13 (0–40)	15 (13–17)	289 (242–323)
	2	III	16 (0–38)	16 (13–17)	292 (245–323)
	3	II	29 (10–51)	16 (12–19)	305 (232–355)
6000	1	III	14 (0–40)	16 (14–18)	300 (255–331)
	2	I	23 (3–47)	16 (14–19)	307 (253–352)
	3	II	32 (9–52)	17 (14–20)	319 (252–368)
6500	1	III	16 (0–43)	17 (14–18)	310 (261–342)
	2	III	22 (4–47)	17 (14–19)	316 (268–350)
	3	III	35 (11–52)	18 (14–20)	333 (267–383)
7000	1	II	21 (1–53)	17 (15–19)	324 (276–359)
	2	III	23 (7–45)	17 (15–19)	322 (276–354)
	3	II	39 (16–55)	19 (15–21)	347 (279–398)
7500	1	III	20 (4–47)	18 (15–19)	330 (284–362)
	2	I	32 (15–53)	18 (15–21)	345 (278–394)
	3	II	42 (21–57)	19 (16–22)	361 (292–412)
8000	1	III	22 (7–49)	18 (16–20)	340 (294–371)
	2	I	35 (17–54)	19 (16–22)	359 (291–407)
	3	II	45 (26–59)	20 (16–23)	376 (307–427)
8500	1	III	24 (10–50)	19 (16–20)	350 (303–383)
	2	I	37 (18–55)	20 (16–22)	372 (308–422)
	3	II	47 (30–61)	21 (17–24)	390 (320–442)
9000	1	III	26 (12–52)	19 (17–21)	360 (313–393)
	2	I	40 (21–57)	21 (17–23)	386 (319–436)
	3	II	50 (34–63)	22 (18–24)	405 (334–457)
9500	1	I	38 (23–59)	21 (17–23)	387 (315–437)
	2	I	43 (25–59)	21 (18–24)	400 (332–451)
	3	I	49 (35–61)	22 (18–25)	413 (346–464)
10,000	1	I	39 (23–60)	21 (18–24)	401 (332–452)
	2	I	45 (29–60)	22 (18–25)	414 (346–466)
	3	I	52 (38–62)	23 (19–25)	427 (358–477)
10,500	1	I	41 (23–62)	22 (19–25)	415 (348–467)
	2	I	48 (32–61)	23 (19–25)	429 (359–480)
	3	I	54 (41–64)	23 (20–26)	441 (370–491)
11,000	1	I	43 (25–63)	23 (19–26)	429 (358–480)
	2	I	50 (35–62)	24 (20–26)	443 (372–495)
	3	I	57 (43–67)	24 (20–27)	454 (381–504)
11,500	1	I	46 (29–64)	24 (20–26)	443 (373–496)
	2	I	52 (38–63)	24 (21–27)	457 (388–510)
	3	I	59 (46–70)	25 (21–27)	468 (393–518)
12,000	1	I	48 (32–65)	24 (21–27)	458 (387–511)
	2	I	54 (41–65)	25 (21–28)	472 (401–525)
	3	I	59 (48–68)	26 (21–28)	484 (404–537)

^a^ The standardized lactation curves in the Norfor-database were employed to predict animal requirement for ECM production through the lactation cycle; ^b^ 1, 2 and 3 refer to code for silages in Table 3; ^c^ I, II and III refer to code for concentrates in Table 3; ^d^ DM: dry matter. Silages 1, 2 and 3 represent a normal range in forage qualities found in the Norwegian cattle production; the combinations of silage and concentrate were determined on the basis of minimum cost when the energy requirements of the animal are met.

**Table 3:** Chemical composition (per kg of dry matter) of silages and concentrates in the NorFor ^a^-database used for the operational models.

Feed Type	Code	Nutritional Value	DM ^b^ (g/kg)	Ash (g)	Crude Protein (g)	Crude Fat (g)	NDF ^c^ (g)	Total Acids (g)	Sugar (g)	Starch (g)	Net Energy for Lactation (MJ)
Silage	1	Very high	332	77	167	39	436	62	92	n.d.	7.0
	2	Medium	325	70	157	35	511	63	53	n.d.	6.1
	3	Low	320	68	150	34	538	64	43	n.d.	5.7
Concentrate ^d^	I	High	879	83	200	59	182	n.d.	n.d.	301	8.0
	II	Medium	873	76	194	52	208	n.d.	n.d.	307	7.7
	III	Low	873	76	182	46	202	n.d.	n.d.	390	7.5

^a^ NorFor: Nordic Feed Evaluation System [8]; ^b^ DM: Dry matter; ^c^ NDF: Neutral detergent fiber; ^d^ Concentrates with high (I), medium (II) and low (III) net energy content were FORMEL Energi Premium 80, FORMEL Elite 80 and FORMEL Favør 80, respectively (Felleskjøpet Agri, Lillestrøm, Norway); n.d.: not determined.

**Table 4:** Evaluation of developed and extant basic models ordered by decreasing CCC.

Model	*n*	Prediction Equation	RMSPE, %	ECT, %	ED, %	ER, %	CCC	r	$C_{b}$
Model 2	36	CH_4_ = −3.01 + 1.19 × DMI − 0.103 × FAs + 0.017 × NDF	13.8	0.2	86.1	13.7	0.703	0.70	1.00
Model 3	36	CH_4_ = 1.13 × DMI − 0.114 × FAs + 0.012 × NDF	13.9	0.1	87.3	12.6	0.694	0.69	1.00
[6]	36	CH_4_ = 1.23 × DMI − 0.145 × FAs + 0.012 × NDF	15.3	3.1	73.1	23.8	0.677	0.69	0.99
Model 1	36	CH_4_ = 4.92 + 1.13 × DMI − 0.118 × FAs	15.0	0.9	82.8	16.3	0.650	0.65	1.00
[7]	36	CH_4_ = 6.80 + 1.09 × DMI − 0.15 × FAs	15.3	0.6	79.3	20.1	0.649	0.65	1.00
[9]	36	CH_4_ = 26.0 + 15.3 × DMI + 3.42 × NDF/10 × 0.05565	13.0	0.0	97.6	2.40	0.611	0.70	0.87
[46]	36	CH_4_ = (38.0 + 19.22 × DMI) × 0.05565	15.6	5.2	89.0	5.80	0.547	0.58	0.95
[9]	36	CH_4_ = [160 + 14.2 × DMI − 13.5 × EE/10] × 0.05565	15.6	14.8	84.0	1.20	0.528	0.60	0.87
[9]	36	CH_4_ = (107 + 14.5 × DMI) × 0.05565	14.8	0.7	99.2	0.00	0.504	0.58	0.87
[47]	36	CH_4_ = (20 + 35.8 × DMI − 0.5 × DMI^2^) × 0.716 × 0.05565	15.4	8.2	90.9	0.90	0.434	0.57	0.76

*n*, number of treatment means; CH_4_, methane (MJ/day); DMI, dry matter intake (kg/day); EE, ether extract content (g/kg dry matter; FAs, fatty acid content (g/kg DM); NDF, neutral detergent fiber content (g/kg DM) if not indicated otherwise; RMSPE, root mean squared prediction error expressed as a percentage of the observed mean and in MJ; ECT, error due to bias, as a percentage of total MSPE; ER, error due to regression, as a percentage of total MSPE; ED, error due to the disturbance, as a percentage of total MSPE; CCC, concordance correlation coefficient; r, Pearson correlation coefficient; $C_{b}$, bias correction factor.

**Table 5:** Operational models: CH_4_ emission factors (kg/year per cow), *Y*_m_ and gross energy intake (GEI), estimated using selected basic models at production levels of 6000, 8000 and 10,000 kg energy corrected milk (ECM) assuming 38.0, 43.5 and 50.0% concentrate share in the rations, respectively.

Model ^a^	CH_4_, kg/Year Per Cow ^b^	*Y*_m_^c^, %	GEI ^d^, MJ/Cow and Day
GEI = 159 + 0.02 × ECM + 1.39 × conc.share			
	6000 kg ECM and 38.0% concentrate share		
*Y*_m(S)_ = 7.11 − 7 × 10^−5^ × ECM − 4.1 × 10^−3^ × conc.share	127.7	6.53	298
*Y*_m(M)_ = 7.65 − 1.1 × 10^−4^ × ECM − 5.4 × 10^−3^ × conc.share	130.2	6.66	298
*Y*_m(N)_ = 7.71 − 1 × 10^−4^ × ECM − 4.4 × 10^−3^ × conc.share	131.5	6.72	298
	8000 kg ECM and 43.5% concentrate share		
*Y*_m(S)_ = 7.11 − 7 × 10^−5^ × ECM − 4.1 × 10^−3^ × conc.share	146.5	6.40	349
*Y*_m(M)_ = 7.65 − 1.1 × 10^−4^ × ECM − 5.4 × 10^−3^ × conc.share	147.8	6.45	349
*Y*_m(N)_ = 7.71 − 1 × 10^−4^ × ECM − 4.4 × 10^−3^ × conc.share	150.6	6.57	349
	10,000 kg ECM and 50.0% concentrate hare		
*Y*_m(S)_ = 7.11 − 7 × 10^−5^ × ECM − 4.1 × 10^−3^ × conc.share	164.5	6.25	401
*Y*_m(M)_ = 7.65 − 1.1 × 10^−4^ × ECM − 5.4 × 10^−3^ × conc.share	163.7	6.22	401
*Y*_m(N)_ = 7.71 − 1 × 10^−4^ × ECM − 4.4 × 10^−3^ × conc.share	168.2	6.39	401

^a^*Y*_m(S)_, *Y*_m(M)_ and *Y*_m(N)_ denotes *Y*_m_ calculated based on GEI (Norfor-database) and CH_4_ production which was predicted using the model from Storlien et al. [7], Model 3 and the model from Nielsen et al. [6], respectively; ^b^ Including 60 day of dry period through inclusion of dry cows in the model for predicting daily CH_4_ production (MJ); ^c^ *Y*_m_, methane conversion factor (% of GEI); ^d^ GEI: gross energy intake.

## Data Availability

Raw data were generated at the Norwegian University of Life Sciences. Derived data supporting the findings of this study are available from the corresponding author [A.S.] on request.

## References

[1] American Meteorological Society (2019). An Information Statement of the American Meteorological Society (Adopted by the AMS Council on 15 April 2019). https://www.ametsoc.org/index.cfm/ams/about-ams/ams-statements/statements-of-the-ams-in-force/climate-change1/.

[2] Hammond K.J., Crompton L.A., Bannink A., Dijkstra J., Yáñez-Ruiz D.R., O’Kiely P., Kebreab E., Eugène M.A., Yu Z., Shingfield K.J. (2016). Review of current in vivo measurement techniques for quantifying enteric methane emission from ruminants. Anim. Feed Sci. Technol..

[3] Hindrichsen I.K., Wettstein H.-R., Machmüller A., Jörg B., Kreuzer M. (2005). Effect of the carbohydrate composition of feed concentrates on methane emission from dairy cows and their slurry. Environ. Monit. Assess..

[4] Shibata M., Terada F. (2010). Factors affecting methane production and mitigation in ruminants. Anim. Sci. J..

[5] Zimmerman P. (2011). Method and System for Monitoring and Reducing Ruminant Methane Production. U.S. Patent.

[6] Nielsen N.I., Volden H., Åkerlind M., Brask M., Hellwing A.L.F., Storlien T., Bertilsson J. (2013). A prediction equation for enteric methane emission from dairy cows for use in NorFor. Acta Agric. Scand. Sect. A Anim. Sci..

[7] Storlien T.M., Volden H., Almøy T., Beauchemin K.A., McAllister T.A., Harstad O.M. (2014). Prediction of enteric methane production from dairy cows. Acta Agric. Scand. Sect. A Anim. Sci..

[8] Volden H., Nes S.K., Sandmo T. (2010). Methane emissions from enteric fermentation in Norway’s cattle and sheep population. Method description. The Norwegian Emission Inventory.

[9] Niu M., Kebreab E., Hristov A.N., Oh J., Arndt C., Bannink A., Bayat A.R., Brito A.F., Boland T., Casper D. (2018). Prediction of enteric methane production, yield, and intensity in dairy cattle using an intercontinental database. Glob. Chang. Biol..

[10] Hollmann M., Powers W.J., Fogiel A.C., Liesman J.S., Bello N.M., Beede D.K. (2012). Enteric methane emissions and lactational performance of Holstein cows fed different concentrations of coconut oil. J. Dairy Sci..

[11] Chung Y.H., He M.L., McGinn S.M., McAllister T.A., Beauchemin K.A. (2011). Linseed suppresses enteric methane emissions from cattle fed barley silage, but not from those fed grass hay. Anim. Feed Sci. Technol..

[12] Patel M., Wredle E., Börjesson G., Danielsson R., Iwaasa A.D., Spörndly E., Bertilsson J. (2011). Enteric methane emissions from dairy cows fed different proportions of highly digestible grass silage. Acta Agric. Scand. Sect. A Anim. Sci..

[13] Willén A. (2011). Methane Production from Dairy Cows; #335. Master’s Thesis.

[14] Yunta B.C. (2010). Methane Production of Dairy Cows Fed Cereals with or without Protein Supplement and High-Quality Silage; #317. Master Thesis.

[15] Van Dorland H.A., Wettstein H.R., Leuenberger H., Kreuzer M. (2007). Effect of supplementation of fresh and ensiled clovers to ryegrass on nitrogen loss and methane emission of dairy cows. Livest. Sci..

[16] Dohme F., Machmueller A., Sutter F., Kreuzer M. (2004). Digestive and metabolic utilization of lauric, myristic and stearic acid in cows, and associated effects on milk fat quality. Arch. Anim. Nutr..

[17] Johnson K.A., Kincaid R.L., Westberg H.H., Gaskins C.T., Lamb B.K., Cronrath J.D. (2002). The effect of oilseeds in diets of lactating cows on milk production and methane emissions. J. Dairy Sci..

[18] Storlien T.M., Adler S., Thuen E., Harstad O.M. Effect of silage botanical composition on greenhouse gas emissions from dairy cows.

[19] Storlien T.M., Nes S.K., Garmo T., Thuen E., Harstad O.M. Effects of maturity of ensiled grass on enteric methane emissions from non-lactating dairy cows offered with two levels of concentrate.

[20] Nes S.K., Garmo T., Chaves A.V., Prestløkken E., Volden H., Iwaasa A.D., Krizsan S.J., Beauchemin K.A., McAllister T.A., Norell L. Effects of maturity of ensiled grass on enteric methane emissions from dairy cows offered with two levels of concentrate.

[21] Kidane A., Øverland M., Mydland L.T., Prestløkken E. (2018). Interaction between feed use efficiency and level of dietary crude protein on enteric methane emission and apparent nitrogen use efficiency with Norwegian Red dairy cows. J. Anim. Sci..

[22] Jonker A., Farrell L., Scobie D., Dynes R., Edwards G., Hague H., McAuliffe R., Taylor A., Knight T., Waghorn G. (2018). Methane and carbon dioxide emissions from lactating dairy cows grazing mature ryegrass/white clover or a diverse pasture comprising ryegrass, legumes and herbs. Anim. Prod. Sci..

[23] Johansen M., Hellwing A.L.F., Lund P., Weisbjerg M.R. (2017). Metabolisable protein supply to lactating dairy cows increased with increasing dry matter concentration in grass-clover silage. Anim. Feed Sci. Technol..

[24] Olijhoek D.W., Hellwing A.L.F., Brask M., Weisbjerg M.R., Højberg O., Larsen M.K., Dijkstra J., Erlandsen E.J., Lund P. (2016). Effect of dietary nitrate level on enteric methane production, hydrogen emission, rumen fermentation, and nutrient digestibility in dairy cows. J. Dairy Sci..

[25] Grandl F., Luzi S.P., Furger M., Zeitz J.O., Leiber F., Ortmann S., Clauss M., Kreuzer M., Schwarm A. (2016). Biological implications of longevity in dairy cows: 1. Changes in feed intake, feeding behavior and digestion with age. J. Dairy Sci..

[26] Grandl F., Amelchanka S.L., Furger M., Clauss M., Zeitz J.O., Kreuzer M., Schwarm A. (2016). Biological implications of longevity in dairy cows: 2. Changes in methane emissions and efficiency with age. J. Dairy Sci..

[27] Alstrup L., Hellwing A.L.F., Lund P., Weisbjerg M.R. (2015). Effect of fat supplementation and stage of lactation on methane production in dairy cows. Anim. Feed Sci. Technol..

[28] Staerfl S.M., Amelchanka S.L., Kälber T., Soliva C.R., Kreuzer M., Zeitz J.O. (2012). Effect of feeding dried high-sugar ryegrass (‘AberMagic’) on methane and urinary nitrogen emissions of primiparous cows. Livest. Sci..

[29] Brask M., Lund P., Hellwing A.L.F., Poulsen M., Weisbjerg M.R. (2013). Enteric methane production, digestibility and rumen fermentation in dairy cows fed different forages with and without rapeseed fat supplementation. Anim. Feed Sci. Technol..

[30] Brask M., Lund P., Weisbjerg M.R., Hellwing A.L.F., Poulsen M., Larsen M.K., Hvelplund T. (2013). Methane production and digestion of different physical forms of rapeseed as fat supplements in dairy cows. J. Dairy Sci..

[31] Moate P.J., Williams S.R.O., Grainger C., Hannah M.C., Ponnampalam E.N., Eckard R.J. (2011). Influence of cold-pressed canola, brewers grains and hominy meal as dietary supplements suitable for reducing enteric methane emissions from lactating dairy cows. Anim. Feed Sci. Technol..

[32] O’Neill B.F., Deighton M.H., O’Loughlin B.M., Mulligan F.J., Boland T.M., O’Donovan M., Lewis E. (2011). Effects of a perennial ryegrass diet or total mixed ration diet offered to spring-calving Holstein-Friesian dairy cows on methane emissions, dry matter intake, and milk production. J. Dairy Sci..

[33] Grainger C., Williams R., Clarke T., Wright A.-D.G., Eckard R.J. (2010). Supplementation with whole cottonseed causes long-term reduction of methane emissions from lactating dairy cows offered a forage and cereal grain diet. J. Dairy Sci..

[34] Beauchemin K.A., McGinn S.M., Benchaar C., Holtshausen L. (2009). Crushed sunflower, flax, or canola seeds in lactating dairy cow diets: Effects on methane production, rumen fermentation, and milk production. J. Dairy Sci..

[35] Odongo N.E., Or-Rashid M.M., Kebreab E., France J., McBride B.W. (2007). Effect of supplementing myristic acid in dairy cow rations on ruminal methanogenesis and fatty acid profile in milk. J. Dairy Sci..

[36] Hindrichsen I.K., Wettstein H.-R., Machmüller A., Kreuzer M. (2006). Methane emission, nutrient degradation and nitrogen turnover in dairy cows and their slurry at different milk production scenarios with and without concentrate supplementation. Agric. Ecosyst. Environ..

[37] Cammell S.B., Sutton J.D., Beever D.E., Humphries D.J., Phipps R.H. (2000). The effect of crop maturity on the nutritional value of maize silage for lactating dairy cows 1. Energy and nitrogen utilization. Anim. Sci..

[38] Wilkerson V.A., Glenn B.P., McLeod K.R. (1997). Energy and nitrogen balance in lactating cows fed diets containing dry or high moisture corn in either rolled or ground form. J. Dairy Sci..

[39] Bates D., Mächler M., Bolker B., Walker S. (2014). Fitting linear mixed-effects models using lme4. arXiv.

[40] St-Pierre N.R. (2001). Invited review: Integrating quantitative findings from multiple studies using mixed model methodology. J. Dairy Sci..

[41] Kutner M.H., Nachtsheim C.J., Neter J., Li W. (2005). Applied Linear Statistical Models.

[42] Bibby J., Toutenburg H. (1977). Prediction and Improved Estimation in Linear Models.

[43] Lawrence I., Lin K. (1989). A concordance correlation coefficient to evaluate reproducibility. Biometrics.

[44] Storlien T.M., Harstad O.M. (2016). Measures in Livestock Production; Potential for Reduction in Emissions of Nitrous Oxide and Enteric Methane from the Milk Dome Population Final Report. Report M-471. https://evalueringsportalen.no/evaluering/tiltak-i-husdyrproduksjonen-potensial-for-reduksjon-i-utslipp-av-lystgass-og-enterisk-metan-fra-mjolkekupopulasjonen-sluttrapport/M471.pdf/@@inline.

[45] Van Es A.J.H. (1975). Feed evaluation for dairy cows. Livest. Prod. Sci..

[46] Charmley E., Williams S.R.O., Moate P., Hegarty R.S., Herd R.M., Oddy V.H., Reyenga P., Staunton K., Anderson A., Hannah M.C. (2016). A universal equation to predict methane production of forage-fed cattle in Australia. Anim. Prod. Sci..

[47] Ramin M., Huhtanen P. (2013). Development of equations for predicting methane emissions from ruminants. J. Dairy Sci..

[48] Hristov A.N., Oh J., Firkins J.L., Dijkstra J., Kebreab E., Waghorn G., Makkar H.P.S., Adesogan A.T., Yang W., Lee C. (2013). Special topics—Mitigation of methane and nitrous oxide emissions from animal operations: I. A review of enteric methane mitigation options. J. Anim. Sci..

[49] Van Lingen H.J., Niu M., Kebreab E., Filho S.C.V., Rooke J.A., Duthie C.-A., Schwarm A., Kreuzer M., Hynd P.I., Caetano M. (2019). Prediction of enteric methane production, yield and intensity of beef cattle using an intercontinental database. Agric. Ecosyst. Environ..

[50] Boadi D., Benchaar C., Chiquette J., Massé D. (2004). Mitigation strategies to reduce enteric methane emissions from dairy cows: Update review. Can. J. Anim. Sci..

[51] Bell M., Eckard R., Moate P.J., Yan T. (2016). Modelling the effect of diet composition on enteric methane emissions across sheep, beef cattle and dairy cows. Animals.

[52] Beauchemin K.A., Kreuzer M., O’mara F., McAllister T.A. (2008). Nutritional management for enteric methane abatement: A review. Aust. J. Exp. Agric..

[53] Johnson K.A., Johnson D.E. (1995). Methane emissions from cattle. J. Anim. Sci..

[54] Toprak N.N. (2015). Do fats reduce methane emission by ruminants?—A review. Anim. Sci. Pap. Rep..

[55] McAllister T.A., Cheng K.J., Okine E.K., Mathison G.W. (1996). Dietary, environmental and microbiological aspects of methane production in ruminants. Can. J. Anim. Sci..

[56] Moe P.W., Tyrrell H.F. (1979). Methane production in dairy cows. J. Dairy Sci..

[57] Jayanegara A., Togtokhbayar N., Makkar H.P., Becker K. (2009). Tannins determined by various methods as predictors of methane production reduction potential of plants by an in vitro rumen fermentation system. Anim. Feed Sci. Technol..

[58] Jaurena G., Cantet J.M., Arroquy J.I., Palladino R.A., Wawrzkiewicz M., Colombatto D. (2015). Prediction of the Ym factor for livestock from on-farm accessible data. Livest. Sci..

[59] Kennedy P.M., Charmley E. (2012). Methane yields from Brahman cattle fed tropical grasses and legumes. Anim. Prod. Sci..

[60] Patra A.K. (2013). The effect of dietary fats on methane emissions, and its other effects on digestibility, rumen fermentation and lactation performance in cattle: A meta-analysis. Livest. Sci..

[61] IPCC (2006). Agriculture, forestry and other land use: Emissions from livestock and manure management. 2006 IPCC Guidelines for National Greenhouse Gas Inventories 4.

[62] Hellwing A.L.F., Weisbjerg M.R., Brask M., Alstrup L., Johansen M., Hymøller Larson M.K., Lund P. (2016). Prediction of the methane conversion factor (Ym) for dairy cows on the basis of national farm data. Anim. Prod. Sci..

[63] Lesschen J.P., van den Berg M., Westhoek H.J., Witzke H.P., Oenema O. (2011). Greenhouse gas emission profiles of European livestock sectors. Anim. Feed Sci. Technol..

[64] Bannink A., Van Schijndel M.W., Dijkstra J. (2011). A model of enteric fermentation in dairy cows to estimate methane emission for the Dutch National Inventory Report using the IPCC Tier 3 approach. Anim. Feed Sci. Technol..

[65] Eugène M., Sauvant D., Nozière P., Viallard D., Oueslati K., Lherm M., Mathias E., Doreau M. (2019). A new Tier 3 method to calculate methane emission inventory for ruminants. J. Environ. Manag..

[66] Kirchgessner M., Windisch W., Müller H.L., Engelhardt V.S., Leonhard-Marek S., Breves G., Giesecke D. (1995). Nutritional factors for the quantification of methane production. Ruminant Physiology: Digestion, Metabolism, Growth and Reproduction.

[67] Bonesmo H., Beauchemin K.A., Harstad O.M., Skjelvåg A.O. (2013). Greenhouse gas emission intensities of grass silage based dairy and beef production: A systems analysis of Norwegian farms. Livest. Sci..

[68] (2020). Climate-Smart Agriculture. https://klimasmartlandbruk.no/klimakalkulatoren/.

